# Molecular orchestration of malate and citrate metabolism: regulatory networks governing organic acid dynamics and fruit quality attributes

**DOI:** 10.1093/hr/uhaf292

**Published:** 2025-10-25

**Authors:** Bei-Ling Fu, Li-Yu Chen

**Affiliations:** College of Horticulture, Fujian Provincial Key Laboratory of Haixia Applied Plant Systems Biology, Haixia Institute of Science and Technology, Fujian Agriculture and Forestry University, Fuzhou 350002, China; College of Horticulture, Fujian Provincial Key Laboratory of Haixia Applied Plant Systems Biology, Haixia Institute of Science and Technology, Fujian Agriculture and Forestry University, Fuzhou 350002, China; College of Agriculture, Key Laboratory of Ministry of Education for Genetics, Breeding and Multiple Utilization of Crops, Fujian Agriculture and Forestry University, Fuzhou 350002, China

## Abstract

In most fleshy fruit, malate and citrate represent the predominant organic acids, serving as key determinants of flavor and nutritional quality. Their concentrations undergo dynamic changes driven by complex biosynthetic pathways and multilayered genetic regulation. Beyond their impact on taste, these organic acids have pleiotropic effects, influencing secondary metabolism and postharvest performance. This review synthesizes current knowledge on the molecular mechanism governing malate and citrate metabolism, including genes responsible for biosynthesis, catabolism, and transport, as well as regulatory networks orchestrated by transcription factors, environmental factors, and phytohormones such as ethylene, abscisic acid (ABA), auxin, gibberellin (GA), and salicylic acid (SA) during fruit development and ripening. We also explored how the dynamics of citrate and malate interact with critical quality attributes, including starch metabolism, textural properties, and postharvest performance, while highlighting domestication-selected genes that influence acidity. Finally, we propose an integrative model delineates the multifactorial regulation of organic acid metabolism in fleshy fruits.

## Introduction

Flavor, a primary organoleptic trait of fleshy fruits, represents a crucial determinant of fruit quality that directly affects consumer preference. Organic acids, alongside sugars and volatile compounds, are crucial soluble components that contribute to the flavor of fleshy fruits [1]. The taste of fleshy fruits is primarily dictated by the balance between total acidity and sugar content [2–4]. Acidity also serves as a critical quality parameter at the time of commercial harvest. Understanding the metabolic pathways and genetic regulations that underlies organic acid accumulation in fleshy fruits is essential for targeted quality improvement and advanced breeding strategies.

In fleshy fruit cells, most organic acids are synthesized intracellularly, with mitochondrial metabolism and vacuolar compartmentation playing key roles in acid accumulation and storage, respectively [5]. These complex regulatory mechanisms are orchestrated by the interplay of genetic determinants and environmental stimuli throughout fruit ontogeny. Recent studies have progressively elucidated the genetic regulation of malate and citrate metabolism through transcriptomic [6–9], metabolomic [10, 11], and proteomic approaches [12, 13], as well as integrated multi-omics analyses of specific cultivars [14–16]. Genome-wide association studies (GWAS) and population genetics approaches have further characterized candidate genes underlying metabolic traits associated with malate and citrate accumulation [17–23]. Nevertheless, the precise regulatory mechanisms governing citrate and malate homeostasis require further elucidation.

Recently, a comprehensive suite of reviews and in-depth studies has thoroughly documented the mechanisms and regulatory networks governing organic acid accumulation in fruit crops [5, 24, 25]. Although the genetic basis, key genes, and regulatory mechanisms have been explored, there remains a lack of systematic syntheses focusing specifically on the conservation or divergence function of transcription factors across species, on responses to environmental stress and phytohormone signals, and on the roles of citrate and malate during domestication. In this review, we profile the main organic acid components across fleshy fruits and synthesize recent advances in understanding the molecular mechanisms regulating malate and citrate dynamics. We critically evaluate candidate genes involved in the metabolism of these organic acids, including biosynthetic enzymes, transporters, and components of degradation pathway. Additionally, we explore the regulatory hierarchies orchestrated by transcription factor networks, environmental factors (temperature, water status, nitrate availability, and salinity stress) and phytohormonal pathways—specifically ABA, ethylene, auxin, GA, and SA signaling—that modulate organic acid accumulation during fruit development and ripening. Our analysis integrates how fluctuations in organic acid interact with key quality parameters such as starch metabolism, textural properties, postharvest performance, and pathogen resistance, revealing an interconnected molecular coordination. Finally, we summarize specific genes central to citrate and malate metabolism that are selected during domestication and functionally shape flavor profiles. This review aims to elucidate the selective domestication processes underlying citrate and malate accumulation in diverse fleshy fruits, and to propose novel strategies for breeding fruit varieties with tailored acid profiles using genetic improvement approaches.

## Diverse organic acid profiles in different fleshy fruits

Fleshy fruits represent a phylogenetically diverse group of species, and the classification based on organic acid content provides a valuable framework for understanding flavor profiles, postharvest physiology, and processing suitability. They contain a broad array of organic acids, such as malic, citric, quinic, succinic, tartaric, oxalic, shikimic, and fumaric acids (Table 1). Among these, malic and citric acids are ubiquitous and contribute to unique organoleptic qualities: citric acid produces a sharp and intense sourness that is rapidly perceived and short lived, whereas malic acid contributes to a refreshingly tartness often accompanied by a mild astringent undertone [26]. Malic acid predominates in several fruit species, including apple (*Malus domestica*) [27], peach (*Prunus persica*) [28], litchi (*Litchi chinensis*) [29], and loquat (*Eriobotrya japonica*) [30]. By contrast, citrus (*Citrus* spp.) [31, 32] and pineapple (*Ananas comosus*) [33, 34] are characterized as the typical citric acid-dominant fruits. Fruits, such as pear (*Pyrus* spp.) [35] and strawberry (*Fragaria* × *ananassa*) [36], accumulate comparable levels of citric and malic acids, resulting in a more complex sourness profile. Some fruits are distinguished by other dominant organic acids; for example, kiwifruit (*Actinidia* spp.) contains substantial quinic acid [37], while tartaric acid predominates in grape (*Vitis vinifera*) [38]. Beyond which acids are present, total acid concentration, the sugar–acid ratio, and interactions with volatile compounds collectively shape perceived flavor. Therefore, organic acid composition serves as an essential biochemical indicator for fruit classification and quality evaluation.

**Table 1:** Content of individual organic acids in different fleshy fruit (mg/g FW)

Species	Major organic acid	Malic acid	Citric acid	Quinic acid	Succinic acid	Tartaric acid	Oxalic acid	Shikimic acid	Fumaric acid	Reference
Apple	Malic acid	3.10–4.6^a^	0.11–0.45^a^	\	0.14–0.51^a^	0.08–0.21^a^	\	\	0.63–1.25^a^	[27]
Peach	Malic acid	2.81–22.88	3.37–6.53	2.81–6.75	\	nd-0.62	0.41–1.47	\	nd-0.05	[28]
Litchi	Malic acid	1.60–3.70	nd	\	\	0.03–0.08	nd	\	\	[29]
Loquat	Malic acid	3.65–8.42	0.06–0.15	\	0.10–0.31	0.41–0.90	0.15–0.27	\	\	[30]
Citrus	Citric acid	2.20–9.00	6.57–48.00	\	1.10–5.40	2.40–8.90	\	\	\	[31, 32]
Pineapple	Citric acid	0.85–1.69	1.13–3.78	0.24–0.93	\	\	\	\	\	[34]
Pear	Malic acid/ Citric acid	0.54–3.91	0.00–8.50	0.007–3.05	\	\	0.04–0.54	0.004–0.29	\	[35]
Strawberry	Malic acid/Citric acid	1.50–3.73	3.52–6.89	\	\	\	\	0.002–0.027	0.005–0.013	[36]
Kiwifruit	Citric acid/ Quinic acid	1.11–3.89	3.42–11.36	4.78–19.85	0.03–0.94	\	0.11–0.82	nd-0.02	\	[37]
Grape	Malic acid/Tartaric acid	1.03–9.68^b^	0.31–2.63^b^^yy^	\	0.11–1.13^b^	1.48–10.99^b^	0.01–0.52^b^	\	\	[38]

\ indicates that there is no clear relevant data in the references.

a represents the value measured in mg/100 ml.

b represents the value measured in g/L.

## What cause the change of malate and citrate content in fruits?

### The enzymes associated with malate biosynthesis and degradation

**Figure 1:**
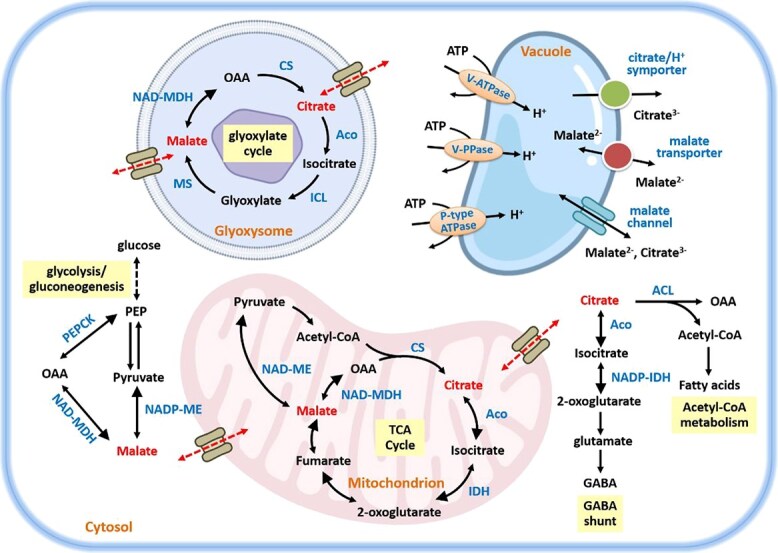
Comprehensive overview of citrate and malate metabolic pathways and transport in fruit cells (modified from Etienne *et al.*, [5]). Aco, aconitase; ACL, ATP-citrate lyase; CS, citrate synthase; ICL, isocitrate lyase; IDH, isocitrate dehydrogenase; MS, malate synthase; NAD-MDH, NAD-malate dehydrogenase; NAD-ME, NAD-malic enzyme; NADP-IDH, NADP-isocitrate dehydrogenase; NADP-ME, NADP-malic enzyme; PEPCK, phosphoenolpyruvate carboxykinase; V-ATPase, vacuolar H^+^-adenosine triphosphatase; V-PPase, vacuolar H^+^-pyrophosphatase. The larger black arrows indicate the favored direction of reversible reactions.

The fluctuation of malate and citrate content in fruit cells is governed by a delicate balance among synthesis, degradation, utilization, and transport. These intricate biological processes involve multiple metabolic pathways (Fig. 1). In the cytosol, malate synthesis primarily occurs through the reversible conversion of oxaloacetate (OAA), catalyzed by NAD-dependent malate dehydrogenase (NAD–cytMDH) [39]. Consistent with this mechanism, the overexpression of the apple *MdcyMDH* gene in apple callus and tomato has been shown to enhance the malate content and upregulate genes related to malate metabolism, indicating its significant role in regulating malate synthesis and accumulation [40]. Further evidence of genetic regulation is provided by a promoter indel in the malate dehydrogenase gene *MdMa7*, which modulates malate content in apple fruit, as observed in studies of hybrids and cultivated varieties [41]. Conversely, reduced NAD–cytMDH activity is a primary factor contributing to the decline of malate during pear storage [42].

Malate degradation primarily occurs via decarboxylation, most likely mediated by phosphoenolpyruvate carboxykinase (PEPCK) and the NADP-dependent cytosolic malic enzyme (NADP–cytME). The role of PEPCK in malate dissimilation has been discussed in several fruit species [43]. The decrease in malate concentration observed across diverse fruits likely reflects the activities and/or abundance of these enzymes [42, 44–46]. In mitochondrial, NAD–mtMDH and NAD–mtME are involved in malate metabolism. The increased activities of NAD–mtMDH and NAD–mtME have been shown to be consistent with the patterns of malate degradation in tomato [47], strawberry [48], loquat [49], and grape [39, 50]. Moreover, malate metabolism can be regulated through the glyoxylate cycle. The expression pattern of malate synthase (MS) correlated with malate accumulation during the development of grape berry [51] and the ripening of banana [52] and tomato fruit [53].

### The enzymes involved in citrate biosynthesis, degradation, and utilization

The enzymes of the TCA cycle play a direct role in catalyzing citrate synthesis and its subsequent interconversion (Fig. 1). Mitochondrial citrate synthase (mtCS) is responsible for most citrate synthesis. In several fruits, mtCS activity increases significantly alongside citrate accumulation [48, 54, 55]. However, some studies have shown that mtCS activity does not account for the differences in citrate levels between high- and low-acid cultivars of citrus [54], peach [56], pineapple [33], and melon [57]. Mitochondrial aconitase (mtAco), which catalyzes the conversion of citrate to isocitrate, is a major determinant of citrate levels. An aconitase inhibitor citramalate caused a threefold increase in citrate levels in lemon fruit [58]. Furthermore, in a low-acid orange mutant, significantly elevated citramalate levels and markedly reduced mtAco activity suggested enhanced citrate synthesis and subsequent export to the cytoplasm [10]. In an introgression tomato line with consistently elevated citrate levels, total aconitase activity and the expression of two *SlAco3* genes were significantly reduced [59]. Following its synthesis, isocitrate is converted to 2-oxoglutarate by isocitrate dehydrogenase (IDH), with both forms, NADP-IDH and NAD-IDH, present in the mitochondrial matrix [60]. A reduction in NADP-IDH activity was found to correlate with the mtAco activity during the early development of lemon fruit [61, 62], suggesting decreased citrate availability within mitochondria.

Once transported into the cytosol, citrate is utilized through two primary pathways: the γ-aminobutyrate (GABA) shunt [63] and acetyl-CoA catabolism via ATP-citrate lyase [64] (Fig. 1). cytAco and NADP–cytIDH are key enzymes in the GABA shunt, with cytAco activity positively correlating with citrate content during pear fruit development [65]. The higher enzymatic activities and transcript levels of *cytAco* and *NADP-IDH* promote cytosolic citrate utilization over vacuolar accumulation [10]. Conversely, low activity of GABA shunt enzymes significantly contributes to high citrate accumulation in high-acid fruits during postharvest storage [32]. ATP-citrate lyase (ACL) catalyzes acetyl-CoA synthesis, and increased *ACL* transcript levels likely respond to declining citrate levels in citrus [66]. Genes within these two pathways were associated with the citrate variation in blueberries [67]. Moreover, *cytAco* and *ACL* exhibit a negative correlation and synergistically control citrate biosynthesis in citrus fruits [68].

### Intracellular carriers of citrate and malate transport

In addition to the biosynthesis and degradation of malate and citrate, vacuolar transporters are essential for the storage of organic acids within fruit cells. Specialized transporters and channels facilitate the movement of malate and citrate across the vacuolar membrane, enabling their exchange at the tonoplast (Fig. 1). The vacuolar transport of malate (a dianion) and citrate (a trianion) occurs via facilitated diffusion, likely through specific malate-permeable channels [69–71]. In Arabidopsis, several aluminum-activated malate transporters (ALMTs) serve as malate specific channels, mediating directional influx or efflux across membranes [72–74]. Extensive studies have shown that ALMTs facilitate the release of malate form vacuoles, directly affecting fruit malate concentrations. In grape berries, VvALMT9 functions as a vacuolar malate channel that mediates malate and tartrate currents [75]. In tomato, *SlALMT9* is the key gene within the *TFM6* locus responsible for variations in malate accumulation. CRISPR-Cas9-induced mutations in *SlALMT9* result in a reduced fruit malate content [17]. Additionally, a polymorphism near *SlALMT9*, specifically in or near *Solyc06g072930.2*, may also influence malate content in this region [19]. In apple, the major quantitative trait locus *Ma* has been identified as the principal regulator of fruit acidity variation. The expression of *Ma1* (an ALMTII-family vacuolar malate channel gene) is significantly correlated with fruit titratable acidity and is recognized as the main determinant of fruit acidity at the *Ma* locus [76, 77]. Its domestication-selected truncated allele, *ma1*, leads to reduced acidity, despite other genetic contributors [78]. Further research indicates *ma1* lacks the conserved C-terminal domain essential for full transporter function, resulting in significantly reduced activity compared to *Ma1* [79]. Overexpression of *Ma1* substantially reduces apple fruit malate content, leading to the discovery of its alternative splicing isoforms, Ma1*α*/*β*, that regulate malate transport [80]. ALMTs have also been suggested to play a role in modulating fruit citrate content in citrus [81]. Additionally, electrophysiological experiments have confirmed that the AcALMT1 protein functions as a citrate transporter in kiwifruit, and transient overexpression of *AcALMT1* specifically increases citrate content in the fruit [9]. Therefore, it is important to distinguish the specific functional members of ALMTs for malate and citrate regulation, particularly in crops rich in diverse organic acids.

In addition to malate-permeable channels, the tonoplast dicarboxylate transporter (tDT) functions at the vacuolar membrane. The Arabidopsis AttDT protein has been well characterized at molecular level. Knockout mutants of *AttDT* exhibit reduced malate levels in leaves [82–84]. Based on sequence homology with *AttDT*, its ortholog *SlTDT* was identified in tomato [85]. Analyses of organic acids content in *SlTDT* overexpression and RNAi transgenic fruits suggest that SlTDT functions as a malate^2−^/citrate^3−^ antiporter, facilitating malate import coupled with citrate export. Similarly, CsCit1, a vacuolar citrate^3−^/H^+^ symporter in citrus and another AttDT homolog, mediates citrate-dependent H^+^ efflux and influences citrate accumulation [86].

Moreover, the activity of proton pumps leads to the uptake of organic acids into the vacuole and is essential for maintaining the electrochemical gradient balance. Vacuolar H^+^-ATPase (V-ATPase, VHA), vacuolar H^+^-pyrophosphatase (V-PPase, VHP), and P-type ATPase are three types of vacuolar proton pumps present in plant vacuoles. The increased activities of V-ATPase and V-PPase were observed during grape ripening, coinciding with a dramatic loss of tonoplast integrity [87]. This suggested that proton pumps contribute to vacuolar acid storage by generating the necessary proton gradient. Furthermore, several studies have provided evidence that differences in citrate or malate accumulation between cultivars may correlate with distinct activity patterns of V-ATPase and V-PPase, such as in peach [56], apple [46], and loquat [49]. In ‘Ponkan’ fruit, the expression of *CitVHA-c4* was associated with higher citrate content [88]. Conversely, reduced expression of some *VHA* and *VHP* genes in a low-acid orange mutant resulted in decreased vacuolar citrate uptake [10]. Interestingly, tonoplast V-ATPase and V-PPase act synergistically in vacuolar acidification, with neither enzyme able to compensate for the loss of the other [89]. In addition, the petunia gene *PH5* encodes a plasma membrane-localized P-type H*-ATPase that controls vacuolar acidity. Using the *PH5* sequence, researchers identified at least eight homologs (*CsPH1* to *CsPH8*) in citrus [90]. Subsequently CsPH4 was confirmed as an R2R3–MYB transcription factor containing the conserved R2R3 repeat domain and C1 and C3 motifs [91]. *CitPH1* and *CitPH5* are required for vacuolar hyperacidification in citrus juice vesicles, conferring their characteristic sour taste. Over millennia of citrus breeding, sweet-tasting varieties emerged recurrently through independent mutations in distinct transcription regulators controlling *CitPH1* and *CitPH5* expression [92]. A decrease in *CsPH8* transcripts lead to low citrate content in low-acid cultivar [90]. In lemon, the P-type ATPase proton pump *AHA10* was undetectable in nonacid fruit but highly expressed in sour varieties [93]. Beyond citrus, the P_3A_-ATPase proton pump gene *Ma10* plays a significant role in regulating malate content in apple fruit. Overexpression of *Ma10* increased malate accumulation in apple callus, and its ectopic expression in tomato lowered fruit pH [94]. Thus, the division of labor among proton pumps in regulating vacuolar acidity warrants further investigation.

Collectively, numerous studies demonstrate that both metabolic pathways and vacuolar transport machineries play crucial roles in the accumulation and degradation of malate and citrate. However, the predominant process responsible for acidity variation within fruit cells remains elusive. Far from being isolated metabolites, malate and citrate serve as central nodes in a dynamic and interconnected network that shifts from anabolic (acid biosynthesis during fruit development) to catabolic (acid degradation during ripening) modes. In Arabidopsis mitochondria, for example, the dicarboxylate carrier DIC2 acts as a high-affinity and directional malate_in_–citrate_out_ antiporter, preferentially exchanging malate for citrate in proteoliposomes [95]. Although a comparable transporter has not yet been identified in fleshy fruits, this potential mechanism provides valuable insight into the tight coupling between citrate and malate that undoubtedly shapes fruit acidity, flavor, and quality. Harnessing high-throughput sequencing and bioinformatics analyses to pinpoint key genes involved in organic-acid synthesis, degradation, utilization, and transport will be instrumental in elucidating the complex networks governing malate and citrate metabolism.

## Transcription factors regulating the variation of malate and citrate levels in fleshy fruit

Over the past decade, a growing body of evidence has underscored the pivotal role of specific transcription factors in orchestrating the expression of genes that define key fruit quality traits, including color development, textural modifications, and the biosynthesis of flavor compounds [96–100]. Building upon this foundation, this review synthesizes and discusses the current understanding of transcription factor functions in the precise regulation of malate and citrate metabolism, with a particular focus on their accumulation and degradation pathways, within fleshy fruits (Table 2).

**Table 2:** Transcription factors reported to possess a citrate or malate regulation-related role in fleshy fruit

Species	Transcription factor	Role	Direct target genes^a^	Regulated genes^b^	Reference
*Solanum lycopersicum*	**SlAREB1**	Increases malate and citrate accumulation in overexpressing line	*n.d.*	*SlmCS、SlgCS、SlmICDH、SlPEPC、* *SlACOH、SlmMDH*	[101, 102]
*S. lycopersicum*	**SlWRKY42**	Controls fruit malate transport and accumulation	*SlALMT9*	n.d.	[17]
*C. sinensis*	**TRY、bHLH35、NAC7、** **bHLH113 (Cs8g04720** **and Cs5g22140)**	Positively or negatively regulate citrate accumulation	*CsAco1、CsAco3*	n.d.	[103]
*C. sinensis*	**CrMYB73 (also** **designated CitPH4)**	Activator of citrate accumulation	*CitTRL*	n.d.	[104, 105]
*C. sinensis*	**CsABF3**	Positively regulates citrate accumulation under drought stress	*CsAN1、CsPH8*	n.d.	[106]
*C. sinensis*	**CsCPC**	negative regulator of citric acid accumulation	*n.d.*	*CsPH1、CsPH5*	[107]
*C. reticulata*	**CitERF6**	Regulates citrate degradation via ATP-citrate lyase	*CitAclα1*	n.d.	[108]
*C. reticulata*	**CitNAC62、 CitWRKY1**	Regulate citrate degradation and decrease citrate content	*CitAco3*	n.d.	[109]
*C. reticulata*	**CitMYB52、CitbHLH2**	Negatively regulate citrate accumulation	*CitALMT*	n.d.	[81]
*Malus domestica*	**MdMYB1、 MdMYB10**	Control cell PH, modulate malate vacuolar transport and increase the accumulation of malate	*MdVHA-B1、MdVHA-B2、MdVHA-E2、MdVHP1、ABC transporter、MdtDT*	*MdVHA-A、MdVHA-B3、MdVHA-D、* *MdVHA-G2、MdmMDH、MdALMT9*	[110]
*M. domestica*	**MdMYB73**	Influences malate accumulation and vacuolar pH	*MdALMT9、MdVHA-A、MdVHP1*	*MdVHA-B1、 MdVHA-B2、MdVHA-B3、 MdtDT、MdPH1、MdPH5*	[111]
*M. domestica*	**MdMYB44**	SNP T/− and SNP A/G in its promoter co-influence malate accumulation	*Ma1、Ma10、MdVHA-A3、MdVHA-D2*	n.d.	[112]
*M. domestica*	**MdMYB123**	Enhances malate accumulation	*MdMa1、MdMa11*	n.d.	[113]
*M. domestica*	**MdMYB21**	Negatively regulates malate accumulation	*MdMa1*	n.d.	[114]
*M. domestica*	**MdbHLH3**	Promotes malate accumulation	*MdcyMDH*	n.d.	[115]
*M. domestica*	**MdWRKY126**	Positively regulates cytoplasm malate anabolism and coordinates the intercompartmental transport to regulate malate accumulation	*MdMDH5*	Malate-associated transporters and proton pump genes	[116]
*M. domestica*	**MdWRKY31、MdERF72**	MdWRKY31 functions as a positive regulator of malate accumulation, while MdERF72 acts as its repressor.	*MdALMT9*	n.d.	[117]
*M. domestica*	**MdESE3**	Enhances fruit acidity and malate accumulation	*MdMa11、MdtDT、MdMDH12*	*MdDTC2*	[118]
*M. domestica*	**MdARF2**	Reduces malate level	*MdcyMDH、MdMATEL1*	n.d.	[119]
*Actinidia chinensis*	**AcNAC1**	Promotes citrate accumulation via citrate transport	*AcALMT1*	n.d.	[9]
*A. chinensis*	**AcSQBP9**	Negatively regulates malate and citrate content	*AcPNAD-MDH1*	n.d.	[120]
*Prunus persica*	**PpNAC1、PpNAC5**	Decreases malate and citrate content	*PpTST1、PpTST2、PpGAD*	n.d.	[121, 122]
*P. persica*	**PpBL、PpNAC1**	Increase malate content	*PpALMT4*	n.d.	[21]
*Pyrus pyrifolia*	**PpABF3、PpWRKY44**	Enhanced salinity-induced malate accumulation	*PpALMT9*	n.d.	[123]
*Ziziphus jujuba*	**ZjWRKY7**	Promotes malate accumulation	*ZjALMT4*	n.d.	[124]
*Z. jujuba*	**ZjbHLH113**	enhances citrate degradation	*ZjACO3*	n.d.	[125]

n.d., not determined; *mCS*, mitochondrial citrate synthase; *gCS*, glioxisomal citrate synthase; *mICDH*, mitochondrial isocitrate dehydrogenase; *PEPC*, phosphoenolpyruvate carboxylase; *ACOH*, aconitate hydratase; *mMDH*, mitochondrial malate dehydrogenase; *ALMT*, aluminum-activated malate transporter; *Aco*, Aconitase; *TRL*, R3-MYB transcription factor TRIPTYCHON-LIKE; *CsAN1*, bHLH transcription factor; *CsPH8*, P3A-ATPase; *Aclα*, ATP-citrate lyase subunit *α*; *VHA*, vacuolar H^+^-adenosine triphosphatase; *VHP*, vacuolar H^+^-pyrophosphatase; *tDT*, tonoplast malate transporter; *Ma1*, Al-activated malate transporter 9; *Ma10*, P_3A_-type ATPase 10; *Ma11*, P_3A_-type ATPase 11; *cyMDH*, cytosolic NAD-dependent malate dehydrogenase; *DTC*, mitochondrial dicarboxylate/tricarboxylate transporter; *MATEL*, toxic compound extrusion protein; *PNAD-MDH*, plastid-localized NAD-dependent malate dehydrogenase; *TST*, tonoplast sugar transporter; *GAD*, glutamate decarboxylase.

a Relationship demonstrated via promoter binding assays.

b Relationship demonstrated via gene expression analysis.

In tomato, *SlAREB1*-overexpressing lines exhibit increased accumulation of citrate and malate, accompanied by the upregulation of genes encoding key metabolic enzymes such as CS, ICDH, PEPC, ACOH, and MDH [101, 102]. SlWRKY42 acts as a negative regulator of fruit malate content by directly binding to the eighth W-box in the promoter of *SlALMT9*, which encodes the primary malate transporter in tomato fruit, thereby suppressing its expression [17].

In citrus fruits, comparative transcriptomics has identified five transcription factors that interact with the promoters of *CsAco1* and *CsAco3* in yeast one-hybrid assays, implicating their involvement in citrate variation [103]. *CrMYB73*, also known as *CitPH4*, has been shown to enhance citrate accumulation when overexpressed in heterologous systems [104]. Additionally, CitPH4 acts as a trans-activator of *CitTRL*, forming a feedback loop that prevents excessive citrate accumulation in the pulp by directly binding to its promoter [105]. Seasonal changes trigger ABA signaling, which upregulates *CsABF3* and activates *CsAN1*, leading to the direct induction of *CsPH8* expression. This regulatory cascade promotes vacuolar citrate accumulation via *CsPH8*-mediated transport, resulting in fruit acidification [106]. Overexpression of the R3-MYB *CsCPC* in citrus callus significantly decreases citrate content and downregulates the expression levels of *CsPH1* and *CsPH5* [107]. CitERF6 has been found to trans-activate the promoter of *CitAclα1*, a key gene in citrate degradation, and its overexpression in transgenic tobacco leaves significantly reduces citrate content [108]. Furthermore, several transcription factors collectively regulate citrate content in citrus through protein–protein interactions. CitNAC62 and CitWRKY1 promote citrate degradation by upregulating *CitAco3* transcripts [109], while the CitMYB52–CitbHLH2 complex synergistically trans-activates *CitALMT* to reduce citrate accumulation [81].

In apple, several members of the MYB family have been identified to regulate malate content. MdMYB1 and MdMYB10 are involved in modulating malate transport and anthocyanin biosynthesis, contributing to changes in cellular pH and organ coloration. MdMYB1 binds to the promoters of *MdVHA-B1* and *MdVHA-B2*, while direct target genes of MdMYB10, which participate in vacuolar malate transport and H^+^-pumping, have also been identified [110]. MdMYB73 modulates malate accumulation and vacuolar pH by directly activating vacuolar malate carrier genes (*MdALMT9*, *MdVHA-A*, and *MdVHP1*), with its molecular function confirmed in transgenic apple callus [111]. MdMYB44 negatively regulates fruit malate accumulation by repressing the promoter activity of malate-associated genes *Ma1*, *Ma10*, *MdVHA-A3*, and *MdVHA-D2* [112]. Two allelic variants (a T/− indel and an A/G SNP) in the *MdMYB44* promoter influence fruit malate content: SNP G and SNP are associated with higher malate content, whereas the A allele and the T allele are associated with lower malate content. Similarly, an A-to-T SNP in the final exon of *MdMYB123* generates a truncated protein (*mdmyb123*). Although both the wild-type MdMYB123 and the truncated proteins bind to the *MdMa1* and *MdMa11* promoters, only the wild-type protein activates transcription, thereby enhancing malate accumulation in apple fruit [113]. Conversely, MdMYB21 binds the *MdMa1* promoter to repress its expression and reduce malate accumulation [114]. MdbHLH3 directly upregulates *MdcyMDH* expression, which encodes an enzyme that mediates cytoplasmic malate synthesis [115]. Similarly, MdWRKY126 binds to the *MdMDH5* promoter and promotes its expression, then enhancing cytoplasmic oxaloacetate (OAA)-to-malate conversion and malate accumulation [116]. Reduced *MdWRKY31* expression downregulates *MdALMT9*, acting both directly and indirectly by alleviating *MdERF72*-mediated transcriptional inhibition [117]. The APETALA2 domain-containing transcription factor MdESE3 activates *MdMa11*, *MdtDT*, and *MdMDH12* transcription through direct binding to the ERE motif within their promoters, promoting malate accumulation in apples fruit by coordinating malate synthesis and transport [118]. The transcriptional repressor MdARF2 downregulates the expression of the malate synthase gene *MdcyMDH* and the malate transporter gene *MdMATEL1*, thereby decreasing malate accumulation in apple fruit [119].

Similar regulatory mechanisms exist in other fruits. In kiwifruit, AcNAC1 transcriptionally activates *AcALMT1* through direct promoter binding. CRISPR-Cas9-mediated mutagenesis of *AcNAC1* reduced kiwifruit citrate accumulation, while malate and quinate levels remained unaltered [9]. AcSQBP9 acts as a transcriptional repressor of the *AcPNAD-MDH1* promoter, both *AcPNAD-MDH1* overexpression and *acsqbp9* mutants resulted in reduced malate and citrate contents in kiwifruit plants [120]. In peach, PpNAC1 and PpNAC5 were found to directly bind and significantly activate the expression level of *PpTST1* (a negative regulator of organic acids) and *PpGAD* [22, 122]. Citrate and malate content decreased in both strawberry fruit with transient overexpression of *PpNAC1*/*5* and transgenic tomato fruit with stable *PpNAC1*/*5* overexpression [121, 122]. Furthermore, PpNAC1 synergized with the NAC transcription factor PpBL to transcriptionally activate *PpALMT4*, promoting malate accumulation. This process is enhanced by a 6.7-kb retrotransposon insertion in the *PpALMT4* promoter, which elevates transcriptional activity and consequently increases malate accumulation [21]. PpWRKY44 and PpABF3 play positive roles in salinity-induced malate accumulation by directly binding to the *PpALMT9* promoter in pear [123]. In jujube, the ZjWRKY7–*ZjALMT4* [124] and ZjbHLH113–*ZjACO3* [125] transcriptional modules mediate the accumulation of malate and citrate, respectively.

**Figure 2:**
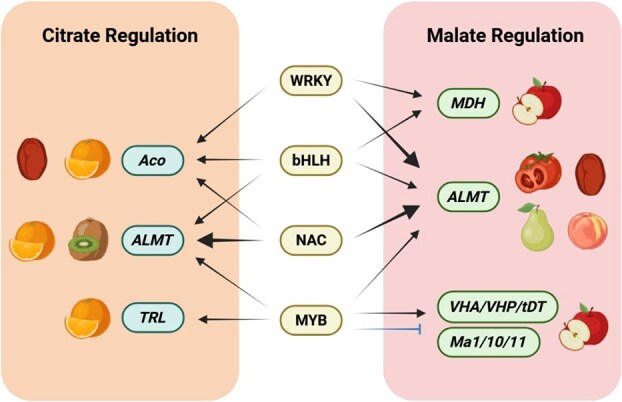
Conserved and species-specific regulatory network controlling citrate and malate accumulation in fruits. Transcription factors including WRKY, bHLH, NAC, and MYB directly regulate genes in citrate and malate metabolism. Arrows indicate activations, while ⊥ indicates repression. Bold arrows indicate this regulatory pathway has been investigated in different species.

Additionally, several transcription factors have been implicated in fruit acidity variation. In tomato, *CgDREB*-overexpressing fruits exhibit increased malate and citrate levels alongside reduced fruit weight, suggesting that CgDREB might acts as a positive regulator of organic acids [126]. Transcriptome analysis identified four transcription factors strongly correlated with acid content as candidate regulators of orange fruit acidity [7]. CitERF13 modulates citrate levels via interaction with the V-ATPase gene *CitVHA-c4* [88]. Although numerous transcription factor families are involved in regulating fruit organic acids, it is evident that transcription factors from divergent families, and even within the same family, exert distinct regulatory roles. Moreover, conserved or species-specific regulatory mechanisms operate across different fruit species (Fig. 2). Most regulatory pathways are species-specific, with transcription factors regulating distinct sets of downstream genes that govern citrate and malate synthesis and degradation. Notably, *ALMT* functions as a hub gene that modulates the accumulation of major organic acids in both citrate- and malate-dominant fruits. The NAC–*ALMT* and WRKY–*ALMT* regulatory pathways appear to be conserved across multiple species. However, in species where citrate and malate accumulate to comparable levels in the fruit, it remains unclear whether ALMT-mediated transport preferentially target one acid or can accommodate both in simultaneously; this question warrants further investigation. Moreover, most identified transcription factors function as activators, while only a few act as repressors in the regulation of organic acids as illustrated. This complexity highlights why the regulatory networks governing malate and citrate dynamics in fleshy fruits remain incompletely characterized. Consequently, both the downstream effectors of these transcription factors and their molecular regulation mechanisms require comprehensive identification and elucidation.

## Environmental drivers of malate and citrate metabolism in fleshy fruits

According to a previous review [5], key environmental factors influencing fruit acidity include the source–sink ratio, mineral nutrition, water supply, and temperature. In recent years, research has increasingly examined on how these environmental conditions shape organic acid accumulation in fleshy fruits. This section summarizes the effects of temperature, water status, nitrate availability, and salinity stress on fruit acidity, with an emphasis on their regulation through phytohormone signaling as well as transcriptional and post-transcriptional control of acid metabolism.

**Figure 3:**
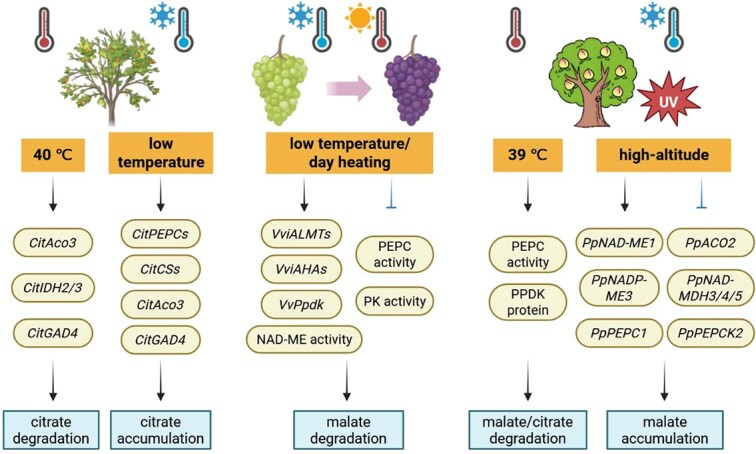
Changes in citrate and malate metabolism under different temperature. The figure summarizes how different temperature regimes regulate organic acid metabolism in fruits (citrus, grape, and peach). It illustrates how temperature-driven shifts in gene expression and enzyme activities lead to changes in citrate or malate levels. Arrows and ⊥ indicate activation and repression, respectively.

Different fruit species display distinct patterns of organic acid variation in response to temperature (Fig. 3). In citrus, exposure to 40°C hot air markedly promoted citrate degradation by up-regulating the *CitAco3*–*CitIDH2/3*–*CitGAD4* cascade [127]. Interestingly, low-temperature treatment also enhanced *CitAco3* and *CitGAD4* expression, which may facilitate citrate accumulation during Ponkan fruit maturation [128]. In grape, malate breakdown was delayed until mid-ripening at 10°C, a pattern correlated with developmental and temperature-dependent expression of specific malate transporters and H^+^-ATPases [129]. Moreover, malate metabolism in grape is regulated differently during day and night. During véraison and ripening, daytime heating accelerated malate degradation and lowered cytosolic pH, in association with increased NAD-ME activity and *VvPpdk* expression level, along with decreased PEPC and PK activities; conversely, warmer nights favored malate retention [130]. Generally, heat treatments reduced both citrate and malate contents, while elevating PEPC activity and PPDK immunoreactive protein levels [131]. In peach grown in high-altitude regions, lower annual mean temperature and stronger ultraviolet radiation increased the expression of organic acid biosynthesis pathway genes (*PpNAD-ME1*, *PpNADP-ME3*, and *PpPEPC1*), as well as degradation-related genes—particularly *PpNAD-MDH2/3/4/5*—thereby promoting malate accumulation [132]. Temperature effects can also interact with other environmental factors; for instance, nitrogen application under high-temperature enhanced citrate and malate accumulation in young tomato fruits by up-regulating *PEPC* expression [133]. Although elevated temperatures generally lead to organic acids accumulation (especially malate) in tomato, accompanied by enhanced PEPC, MDH, and CS activities, adjusting relative humidity to 70% can mitigate high stress and improve fruit quality [134]. These findings provide valuable guidance for optimizing fertilization and cultivation strategies under global warming scenarios.

**Figure 4:**
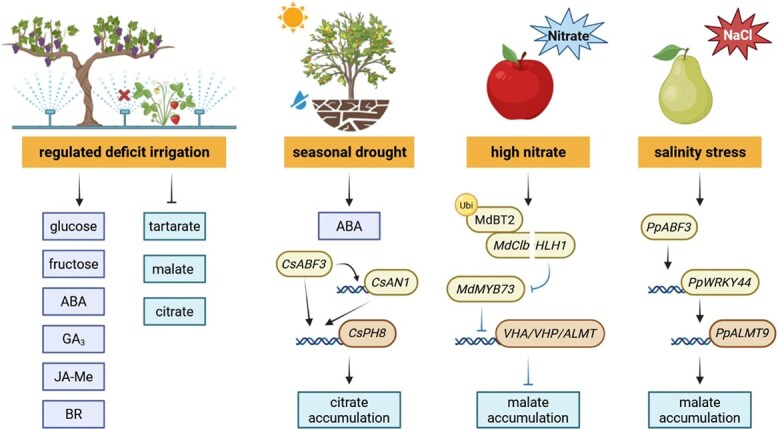
Modulation of citrate and malate metabolism by water deficit, nitrate availability, and salinity stress. The figure summarizes stress-dependent changes in organic acids, sugars, and hormone levels in grape, strawberry, citrus, apple, and pear, along with associated shifts in gene expression, transcriptional regulation, and ubiquitination that alter citrate or malate accumulation. Arrows and ⊥ indicate activation and repression, respectively.

Water availability also exerts a pronounced influence on organic acid dynamics during fruit maturation (Fig. 4). In grape, deficit irrigation reduced tartrate, malate, and citrate contents, and was associated with marked shifts in endogenous hormone levels—increased ABA alongside decreased IAA, GA3, JA-Me, and BR [135]. A similar pattern was observed in strawberry, where deficit irrigation significantly lowered citrate and malate levels, while elevating ABA concentration in secondary fruit [136]. In citrus, drought-induced citrate accumulation is partly attributed to altered *CsPH8* transcript abundance [137]. A subsequent study revealed that seasonal drought during the fruit enlargement stage enhanced citrate accumulation through the CsABF3–*CsAN1*–*CsPH8* module, mediating ABA signaling [106]. Moreover, in jujube fruits, temperature and drought effects interact significantly: elevated temperature reduced organic acid content, whereas concurrent drought stress at the same temperature increased it [138].

Nitrate is an essential macronutrient for plant growth and development, and also acts as a signaling molecule that regulates numerous physiological processes [139]. However, excessive nitrate application can markedly increase citrate and malate concentrations in apricot [140], while reducing fruit acidity in pineapple [141], cherry tomato [142], and apple [143]. In apple, molecular studies have shown that nitrate lowers malate level via the ubiquitination activity of MdBT2, which targets the transcription factor MdCIbHLH1 for degradation, thereby transcriptionally down-regulating several malate-associated genes and inhibiting malate accumulation [143]. In addition, salinity, a major environmental stressor that threatens global crop productivity and sustainable agriculture, has been reported to stimulate the accumulation of organic acids and alter fruit acidity profiles. For instance, salt stress elevates tartrate and malate contents in grape, accompanied by noticeable declines in aroma and taste quality [144]. Similarly, the PpABF3–*PpWRKY44*–*PpALMT9* pathway positively contributes to salinity-induced malate accumulation in pear [123]. In apple, the protein kinase MdSOS2L1 modulates malate levels by phosphorylating MdVHAB1 at Ser^396^, under both salt stress and normal conditions [145]. Collectively, current evidence indicates that fruit organic acids respond in highly species- and context-dependent ways to environmental factors, often involving phytohormone-mediated pathways. Despite these advances, the beneficial roles of environmental factors in shaping the quality parameters of commercially important crops remain underexplored. The molecular mechanisms by which fruit organic acid metabolism responds to external environments arise from a complex regulatory network and remain to be fully elucidated. Together, these studies provide novel insights into how growth environments influence fruit quality attributes.

### Dissecting hormonal control: relative roles in malate and citrate accumulation

Across diverse taxa, malate and citrate are predominant organic acids throughout the development and ripening in both climacteric and nonclimacteric fruits (Table 1). Despite this prevalence, their temporal accumulation patterns vary markedly among species. Notably, the trajectories of malate and citrate concentrations do not consistently correlate with climacteric status (ethylene-mediated ripening) or respiratory intensity, which acid homeostasis is governed by complex, species-specific regulatory mechanisms largely independently of respiratory pathways. The development and ripening of fleshy fruits are orchestrated by sophisticated hormonal regulatory network, primarily involving ethylene, ABA, auxin, cytokinin, GAs, and brassinosteroids (BRs) [100, 146–149]. While substantial evidence highlights the influence of hormones on fruit maturation processes such as sugar accumulation, pigmentation, cell wall hydrolysis, and the biosynthesis of flavor/aroma compounds [146, 148], there is a paucity of studies focusing specifically on hormonal regulation of citrate and malate metabolism during ripening. Recent advancements in omic technologies have enabled the detailed characterization of phytohormone–metabolite interactions during ripening [8, 150–153], paving the way for a deeper understanding of hormonal mechanisms governing organic acid dynamics.

Ethylene plays a pivotal role in regulating the biochemical and signaling pathways that determine organoleptic traits during fruit development in both climacteric and nonclimacteric species [154–156]. The tomato, a well-established model for studying fleshy fruit ripening, benefits from well-characterized mutants that dramatically alter ripening processes. An integrated analysis combining transcriptomic, proteomic, and targeted metabolomic approaches was employed to examine developmental stages in three tomato mutants: ripening inhibitor (*rin*), non-ripening (*nor*) and never-ripe (*Nr*) [150]. This study revealed strong correlations between groups of metabolites (including organic acids, sugar phosphates, and cell wall-related metabolites) and ripening-associated transcripts, underscoring the functional importance of these metabolites. Moreover, studies indicate that TCA-cycle organic acids are significantly correlated with ethylene-responsive genes, suggesting transcriptional regulation of TCA-cycle intermediates [151, 157]. During postharvest ripening, peaches exhibit substantial ethylene production, which coincides with increased citrate, glucose and fructose content [158]. In kiwifruit, metabolite mapping revealed that concentrations of citrate, malate, and sucrose were substantially lower in fruit subjected to exogenous ethylene-induced ripening than in naturally ripened fruit, although these differences did not markedly affect taste variation [159]. Moreover, ethylene regulates ripening in the non-climacteric fruits like strawberry in a tissue- and stage-specific manner. Introducing the ethylene receptor allele *etr1–1* in strawberry reduced ethylene sensitivity and altered fruit ripening, leading to differential regulation of citrate, malate, glucose, fructose, and amino acid metabolites in both the achene and the receptacle [160]. The reduction malate accumulation during climacteric fruit ripening has long been associated with increased ethylene production. The WRKY31–ERF72 complex inhibits the expression of *MdALMT9* in response to ethylene, thereby decreasing vacuolar malate storage [117].

Extensive research has demonstrated the pivotal role of ABA in the ripening of tomato and strawberry fruits, where it modulates fruit softening, anthocyanin biosynthesis, and sugar accumulation by regulating ABA biosynthesis genes, ABA receptors, and transcription factors [161–165]. In tomato, the ABA-response element binding factor SlAREB1 regulates sensory attributes. Notably, levels of citrate, malate, glucose, and fructose are significantly increased in *SlAREB1*-overexpressing lines compared to wild-type and antisense suppression lines. This modulation of organic acids and hexoses is associated with the upregulated expression of genes encoding enzymes involved in TCA cycle and carbon metabolism, highlighting the role of ABA-regulated transcription factors in fruit quality control [101, 102]. In citrus, ABA signaling significantly induces the expression of *CsABF3*, *CsAN1*, and *CsPH8*. The ABA–CsABF3–CsAN1–CsPH8 regulatory module mediates citrate accumulation under drought stress [106]. Additionally, exogenous ABA application upregulates *MdARF2* expression while suppressing *MdcyMDH* and *MdMATEL1*, thereby reducing malate levels. Silencing *MdARF2* abolishes the ABA-mediated suppression of these two key genes, confirming MdARF2 as a central regulator in ABA-dependent malate homeostasis [119].

The roles of crosstalk among auxin, gibberellin, and cytokinin in fruit set initiation have been well characterized [148, 166–168]. The application of these hormones can trigger fruit development. Recently, significant progress has been made in understanding how organic acids interact with auxin-mediated regulation. Exogenous application of auxin has been found to downregulate genes involved in the TCA cycle and oxidative phosphorylation pathways, suggesting that auxin may inhibit tomato fruit ripening by suppressing respiration rates. Moreover, exogenous auxin application altered the expression levels of ethylene –synthesis-related genes, indicating potential modulation via auxin–ethylene interactions [169]. Subsequent primary metabolite profiling of auxin-treated fruits revealed significantly higher levels of citrate, succinate, and citramalate compared to control fruits, highlighting a role for auxin in enhancing fruit acidity [170]. Changes in organic acids are also linked to GAs in fruit. Reduced activities of TCA cycle enzymes, such as malate dehydrogenase and fumarase, have been associated with decreased GA levels in tomato roots [171]. Similarly, in 2-oxoglutarate dehydrogenase antisense tomato fruit, the expression of GA biosynthetic pathway transcripts was reduced, accompanied by decreased GA levels and associated with early leaf senescence and maturation [172]. Recent proteomic analysis revealed that differentially expressed proteins in GA-treated triploid loquat fruits were enriched in the citrate cycle pathway [173]. Furthermore, exogenous application of SA and its derivatives, methyl salicylate (MeSA) and acetyl salicylic acid (ASA), in sweet berries has been shown to delay postharvest ripening and maintain fruit quality attributes such as acidity, firmness, and color [174–176].

Although the regulatory roles of hormones, such as cytokinin and BRs in fruit set, growth, and maturation, have been characterized, the relationship between these hormones and organic acid metabolism remains unclear. To date, relatively few studies have elucidated the precise molecular mechanisms by which hormones regulate malate and citrate metabolism. Altering hormone levels can induce changes in primary metabolites, such as organic acids, demonstrating their potential impact on citrate and malate metabolism. Consequently, manipulating these hormones via application or inhibition represents a direct approach to investigate their roles in controlling fruit quality, offering a promising avenue for further research.

### Malate and citrate metabolism is linked to other key traits throughout fruit development and ripening

In the previous sections, we discussed the roles of metabolism-related genes, transcription factors, and hormones in regulating citrate and malate content. Understanding the connections between malate/citrate metabolism and other critical traits during fruit development and ripening is crucial. Research using established genetic engineering techniques in tomatoes has demonstrated that changes in organic acid metabolism, particularly within the TCA cycle, are linked to various physiological processes. For instance, antisense inhibition of the mitochondrial malate dehydrogenase and fumarase in tomatoes resulted in altered root biomass and growth, with corresponding changes in malate and citrate levels observed in the transgenic lines [171]. Similarly, aconitase antisense transgenic tomato plants exhibited dramatically reduced respiration, root growth, and modifications in cell wall composition, highlighting the role of the TCA cycle in regulating root metabolism and secondary cell wall biosynthesis [177]. Metabolic engineering of transgenic tomato fruit expressing an antisense aconitase construct further demonstrated that malate and citrate accumulation levels are closely associated with key metabolic regulators [59]. Moreover, transgenic tomato plants deficient in the 2-oxoglutarate dehydrogenase complex exhibited remarkably altered metabolic profiles, characterized by lower malate and fumarate but higher citrate levels, along with reduced respiration rates and specific developmental changes, including accelerated flowering, early fruit ripening, and leaf senescence [172].

Malate is also a key regulatory metabolite in transitory starch metabolism [157]. By independently silencing malate dehydrogenase and fumarase in a fruit-specific manner, they demonstrated that changes in malate levels profoundly impact tomato fruit soluble sugar content, postharvest shelf life, and bacterial resistance. Supporting these findings, alterations in the malate–pyruvate interconversion significantly influence starch biosynthesis [45]. The *CitPH4*-knockout mutant exhibits an acidless phenotype with significantly reduced resistance to citrus diseases. In natural citrus populations, the contents of defense-related metabolites (pipecolic acid and quercetin) were positively correlated with pulp citrate levels. This positions CitPH4 as a dual regulator of both disease resistance and fruit acidity, offering a novel strategy for simultaneously improving both traits in citrus breeding [178]. Additionally, exogenous GABA treatment significantly increases citrate and ATP content, which is a probable mechanism for maintaining postharvest quality and enhancing postharvest storage performance [179].

### The metabolic tuning of fruit acidity through domestication syndrome

**Figure 5:**
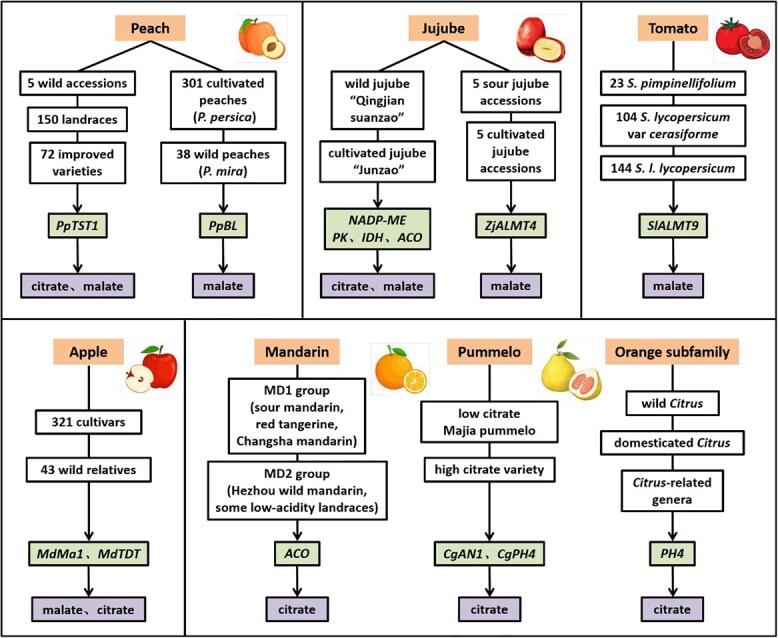
Key genes in citrate and malate domestication during fruit evolution. The figure illustrates the screening of key citrate and malate domestication genes via genome sequencing of wild-cultivated pairs or acidity-variant accessions in fruit crops. The elements in each schematic diagram are arranged from top to bottom, corresponding to fruit species, plant materials, key genes, and domestication-affected organic acids.

Whole-genome resequencing of fruit crops provides comprehensive genomic information for the species. Comparative analysis of genomic variations across diverse accessions allows for the assessment of evolutionary divergence and sheds light on their origins and domestication history. Acidity, a key factor in the evolution of fruit flavor, has undergone significant modifications during domestication. Specific genes involved in the metabolism of citrate and malate, which have been subjected to selection and play a crucial role in flavor development, have been identified (Fig. 5).

In peach, combined GWAS and BSA approach pinpointed a key genomic interval regulating total organic acid content to the 0.47–1.48 Mb region at the proximal end of chromosome 5. Within this interval, the critical gene *PpTST1* was identified. A single-base transversion (G1584T) was detected in its third exon results in two allelic variants, *PpTST1^His^* and *PpTST1^Gln^*. Analyzing the frequency variation and geographical distribution of these two genotypes across 227 accessions revealed strong positive selection for *PpTST1* during peach evolution, aligning with human selection for organoleptic properties that enhance fruit flavor [22]. Complementary studies identified the *PpBL* gene within a significant malate-associated locus on chromosome 5 through GWAS of 301 cultivated peach accessions, suggesting that this locus also underwent selection during peach domestication [21].

During the domestication of jujube, fruit acidity decreased while sweetness increased under human selection [180, 181]. Key genes controlling citrate and malate metabolism within selective sweep regions were identified as *NADP-ME*, *PK*, *IDH*, and *ACO*. These regions encompassed 1372 genes in the jujube genome [180]. Further analysis of selective-sweep data between sour jujube (*Ziziphus spinosa*) and cultivated jujube (*Z. jujuba*) highlighted *ZjALMT4* as a candidate gene associated with malate content, primarily due to three genotypes of the W-box in its promoter [124]. Similarly, during tomato domestication, *SlALMT9* is believed to be responsible for increased malate content [17]. In apple, selective sweeps were detected for reduced fruit acidity, but not for increased sugar content, during both domestication and genetic improvement [182]. This may be due to strong impact of acidity on fruit taste and flavor. The selection of *MdTDT* alongside *Ma1* appears to drive significant changes in fruit citrate and malate composition and acidity. Moreover, the vacuolar acidification-related gene *PH4* was co-located with a fruit weight QTL on chromosome 15, a region identified in the selective sweeps of wild species and heirlooms [183].

In *Citrus*, wild accessions typically exhibit higher fruit citrate content than cultivated varieties. Modern citrus domestication pathways reveal that acidless phenotypes often result from large retrotransposons insertions or deletions within the *Noemi* gene. This allele persists in *Citron* (*Citrus medica*, the first citrus species)-derived hybrids with long cultivation histories [184]. During the domestication of mandarin (*C. reticulate*), a high divergence region in an aconitase (*ACO*) gene was implicated in citrate regulation, possibly under selection [185]. Genomic analyses further identified extensive relatedness among mandarins and sweet orange (*C. sinensis*), shedding light on their domestication patterns. Widespread pummelo (*C. maxima*) admixture in these mandarins correlates with fruit size and acidity, suggesting pummelo introgression facilitated the selection of palatable traits [186]. In pummelo, tissue-specific citrate accumulation in pulp is driven by high *CgAN1* and *CgPH4* expression. Differential methylation of the *CgAN1* promoter in high- versus low-acid pericarps implies epigenetic modifications influenced pummelo domestication [187]. Building on these findings, a recent study collected 314 orange subfamily germplasm genomes globally, performed *de novo* assembly of 12 representative species, and constructed an orange subfamily pangenome. This resource confirms the ancient Indian plate as the origin center for Citrus-related genera, while South-Central China represents the origin center for the Citrus genus. Notably, the *PH4* gene displays significant sequence and expression variations of across the orange subfamily. CRISPR-Cas9 mediated editing of *PH4* in the early-flowering citrus drastically reduced fruit citrate content, affirming its core role in citrate accumulation [188].

Additional studies on the domestication of fruit total acidity have also been reported. In melon (*Cucumis melo*), the *CmPH* gene determines fruit acidity through a four amino acid duplication insertion in nonacidic accessions, contributing to the evolution of sweet melon [189]. This locus was subsequently detected within association signals from a GWAS for flesh acidity and resides in a selective sweep region [190]. Population-scale genotyping of structural variants between wild and cultivated melons with divergent acidity confirmed the key role of *CmPH* in acidity regulation during domestication and selection [191]. Separately, a model for the divergence, dissemination, and domestication of Asian and European pears was proposed [192]. Notably, 14 genes mapped to acidity-related QTLs are implicated in regulating flavor traits selected during pear domestication.

Together, these studies shed light on the fundamental mechanisms of citrate and malate metabolism and unveil selection signatures that have emerged during the domestication of fruit crops. These advancements greatly enhance our understanding of evolutionary biology and crop genomics, offering both theoretical insights and practical applications. By pinpointing key molecular targets for breeding, they facilitate the strategic utilization of germplasm resources, thereby accelerating the development of novel cultivars with optimized fruit acidity—a critical trait influences flavor profiles, postharvest quality, and consumer preferences in global horticultural markets.

### Conclusions and future perspectives

As summarized in this review, our understanding of the regulatory enzymes, transcription factors, and phytohormones that control malate and citrate accumulation in fruits has advanced significantly. Although malate and citrate are the predominant organic acids in fruits, their content vary widely across species (Table 1). While fruit acidity has likely been shaped in part by artificial selection, differences in organic acid composition cannot be attributed to selection alone. For example, malate is consistently present in cultivated apples and dominates in their main wild ancestors, yet high citrate levels occur in 53% of wild apples, indicating that divergence in organic acid profiles reflects underlying genetic architecture and direct genetic effects rather than domestication pressure alone [193]. Together, these observations imply that organic acid metabolism is under complex genetic control and may involve distinct selection mechanisms governing their metabolic pathways and accumulation patterns.

**Figure 6:**
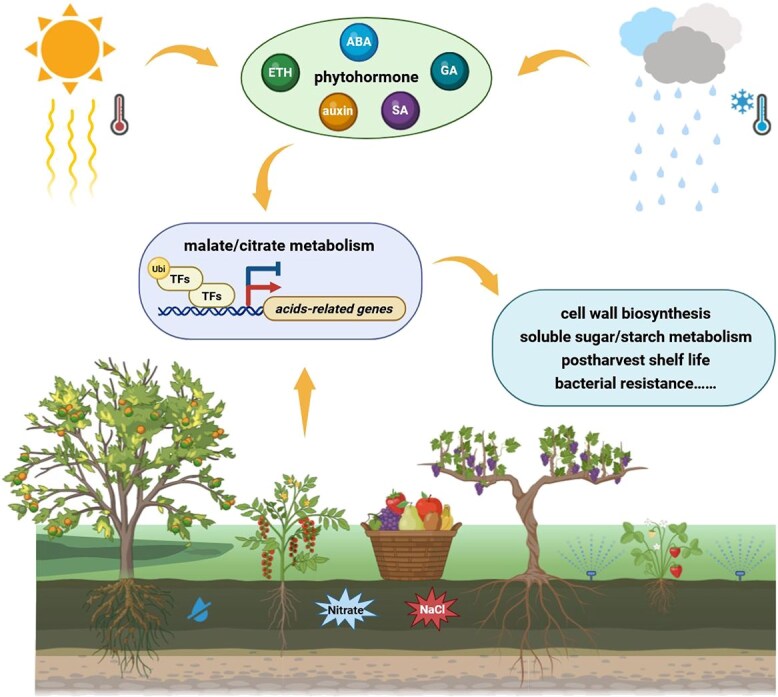
Model of the multifactorial regulation of malate and citrate metabolism during fleshy fruit ripening. Environmental factors, including temperature, water status, nitrate availability, and salinity stress strongly influence acidity throughout fruit development and ripening. These factors also modulate phytohormone signaling, which subsequently regulates the expression of genes governing malate and citrate metabolic pathways. Consequently, they further impact fruit quality attributes such as texture, flavor, postharvest shelf life, and bacterial resistance.

Modern bioengineering approaches have revealed a close connection between organic acid metabolism and other key traits that influence fruit growth and ripening. Environmental factors and phytohormone also modulate citrate and malate accumulation via distinct cellular mechanisms. Building on this foundation, we propose an integrated model that outlines the multifactorial regulation of malate and citrate metabolism during the development and ripening of fleshy fruits (Fig. 6). Although much of current evidence derives from a limited set of species, emerging tools, such as high-resolution genomics and pan-genomics, GWAS/eQTL mapping, single-cell and spatial multi-omics, metabolic flux analysis, and precision genome editing (including base/prime and multiplex editing), now enable systematic dissection of the genetic–metabolic networks controlling acidity across a wide range of fleshy fruits. Further work should resolve the molecular interactions within these networks, quantify genotype-by-environment and hormone interactions, and link causal variations to flux-controlling steps and transport process. These advances will deepen our understanding of fruit flavor evolution and development, and actionable targets for breeding and genome editing to fine-tune acidity and related organoleptic and nutritional attributes without compromising agronomic performance.

## Data Availability

No datasets were generated or analyzed during the current study.
